# Simultaneous Pancreas-Kidney Transplant Complicated by Kidney Allograft Torsion and Pseudoaneurysms of the *Y*-Allograft: A Case Report and Review of the Literature

**DOI:** 10.1155/2022/1748141

**Published:** 2022-12-10

**Authors:** Sarah L. Tan, Rachel Y. P. Tan, Gabrielle Cehic, Michael Wu, John Kanellis, Jeffrey Barbara

**Affiliations:** ^1^Department of Nephrology, Flinders Medical Centre, Flinders Drive, Bedford Park, South Australia 5042, Australia; ^2^College of Medicine and Public Health, Flinders University, Sturt Road, Bedford Park, South Australia 5042, Australia; ^3^Department of Nuclear Medicine, Flinders Medical Centre, Flinders Drive, Bedford Park, South Australia 5042, Australia; ^4^Centre for Translational Cancer Research, University of South Australia (Cancer Research Institute), 101 Currie Street, Adelaide, South Australia 5001, Australia; ^5^Department of Surgery and School of Clinical Sciences, Monash Health, 246 Clayton Road, Clayton, Victoria 3168, Australia; ^6^Monash University, Wellington Road, Clayton, Victoria 3800, Australia; ^7^Department of Nephrology, Monash Health, 246 Clayton Road, Clayton, Victoria 3168, Australia; ^8^Centre for Inflammatory Diseases and Department of Medicine, Monash University, Wellington Road, Clayton, Victoria 3800, Australia

## Abstract

**Background:**

We report and review the literature of two rare complications of simultaneous pancreas-kidney transplantation (SPKT) occurring in one patient. *Case Report*. A 39-year-old man with dialysis-dependent kidney failure secondary to type 1 diabetes mellitus underwent successful SPKT in October 2018. Three months later, he presented with an acute kidney injury (AKI) and returned to dialysis. Kidney scintigraphy showed a central photopenic region, and angiograms showed absent flow in the kidney transplant artery without treatable thrombus and the incidental finding of two pseudoaneurysms of the pancreatic *Y*-graft. He remained dialysis-dependent for three weeks before spontaneous partial recovery of allograft function; repeat kidney scintigraphy showed significant improvement in perfusion. However, in April 2019 he was readmitted with a sudden deterioration in kidney allograft function again necessitating haemodialysis. Repeat imaging confirmed that the kidney allograft had shifted from the left iliac fossa to the midline. He underwent surgical exploration, during which torsion of the kidney allograft was confirmed and a nephropexy was performed. The kidney allograft was originally implanted in the left retroperitoneum via a midline transperitoneal approach, which likely predisposed it to torsion. The pseudoaneurysms of the pancreatic *Y*-graft were managed conservatively, and surveillance imaging demonstrated that they remained stable in size. The patient regained reasonable kidney allograft function (estimated glomerular filtration rate, eGFR, of 45 mL/min) and maintained normal pancreatic allograft function.

**Conclusion:**

Kidney allograft torsion should be considered post-SPKT in patients with AKI and absent arterial flow. Although most case reports describe surgical management of pseudoaneurysms post-SPKT, our case demonstrates successful conservative management.

## 1. Introduction

SPKT is an established treatment option for patients with type 1 diabetes who are dialysis-dependent. There were 38 SPKT recipients in Australia in 2019 [1]. Although SPKT offers numerous benefits, including improved patient survival; recipients tend to experience surgical complications at a relatively higher rate in comparison to kidney-only recipients, without impact on longer-term allograft function [2–4].

We describe and review the literature of two rare complications of SPKT occurring in the same patient: torsion of the kidney allograft and pseudoaneurysms of the pancreatic *Y*-graft.

## 2. Case Presentation

A 39-year-old man with kidney failure secondary to type 1 diabetes mellitus received a simultaneous pancreas-kidney transplant in October 2018 after four years on haemodialysis. Via a transperitoneal approach, the donor's left kidney was placed in the left iliac fossa. The single transplant renal artery was anastomosed end-to-side to the left external iliac artery; the single transplant renal vein was anastomosed to the left external iliac vein, and the transplant ureter to the recipient's bladder. The donor pancreas was vascularised via a *Y*-graft from the donor splenic and superior mesenteric arteries to the recipient's right common iliac artery; the donor portal vein anastomosed to the recipient's inferior vena cava. The donor duodenum was anastomosed to the recipient's jejunum for exocrine drainage.

Postoperatively, he returned to theatre on day 0 post-transplant. A repeat laparotomy demonstrated bleeding around the venous anastomosis of the kidney allograft and a small branch off the inferior vena cava (IVC), with haemostasis being achieved.

The patient made an excellent recovery and was discharged two weeks post-transplant on triple immunosuppressive therapy: tacrolimus, mycophenolate, and prednisolone. Prior to discharge, he was euglycaemic with a creatinine of 94 *μ*mol/L and lipase of 33 U/L. A contrast computed tomography (CT) scan performed on day 22 post-SPKT showed patent transplant vessels of both the pancreatic and kidney allografts with no abnormalities of the vasculature. No abnormal positioning of either allograft was noted.

**Figure 1:**
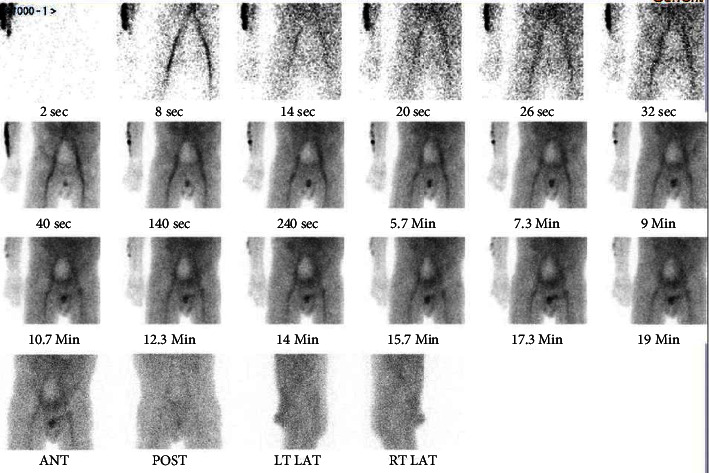
Kidney scintigraphy on day 95 post-transplantation demonstrating a central photopenic region, suggesting absent perfusion of the kidney allograft.

He presented three months later (day 95 post-transplant) with AKI - creatinine 403 *μ*mol/L and lactate dehydrogenase of 2301 U/L. He was asymptomatic. Three days prior to admission, his creatinine had been 104 *μ*mol/L. He remained euglycaemic with a normal lipase. Kidney scintigraphy showed a central photopenic region (Figure 1), and subsequent angiography confirmed minimal flow in the transplant renal artery with a nonperfused kidney allograft and no thrombus amenable to intervention. Torsion of the vessels was not observed at the angiogram. A further contrast CT scan demonstrated no differential enhancement of the transplant kidney between the arterial and portal venous phases, and the diagnosis was presumed kidney allograft loss secondary to infarction. The placement of the kidney allograft was not commented on.

**Figure 2:**
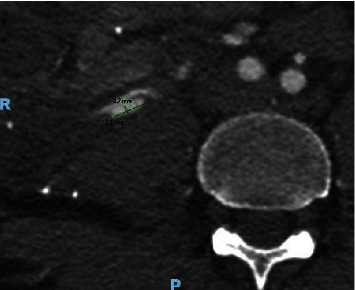
Contrast computed tomography scan of the abdomen showing one of the two pseudoaneurysms of the superior artery of the *Y*-graft.

The CT also revealed the development of two pseudoaneurysms of the superior iliac artery of the *Y*-graft (Figure 2) measuring 12 × 4 mm and 3 mm, respectively.

Extensive investigation looking for infection and vasculitis (including blood cultures, antineutrophil cytoplasmic antibodies, antinuclear antibody, extractable nuclear antigen antibodies, and an antiphospholipid antibody screen) as potential causes of the pseudoaneurysms returned negative results and a decision was made to opt for conservative management. The creatinine peaked at 766 *μ*mol/L and he was re-established on haemodialysis and discharged to home.

**Figure 3:**
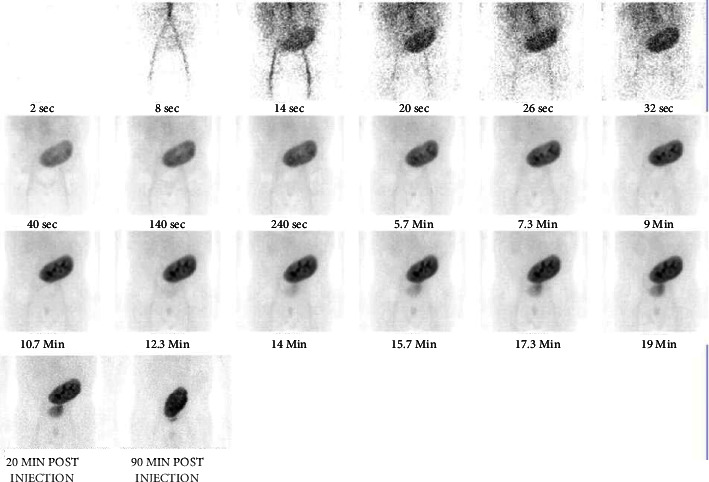
Kidney scintigraphy performed on day 115 post-transplantation demonstrating a return of perfusion to the kidney allograft.

The patient underwent 11 sessions of haemodialysis over 3 weeks. A repeat kidney scintigraphy was performed after he reported increasing urine output and this revealed improvement in kidney perfusion with no evidence of vascular insult (Figure 3).

**Figure 4:**
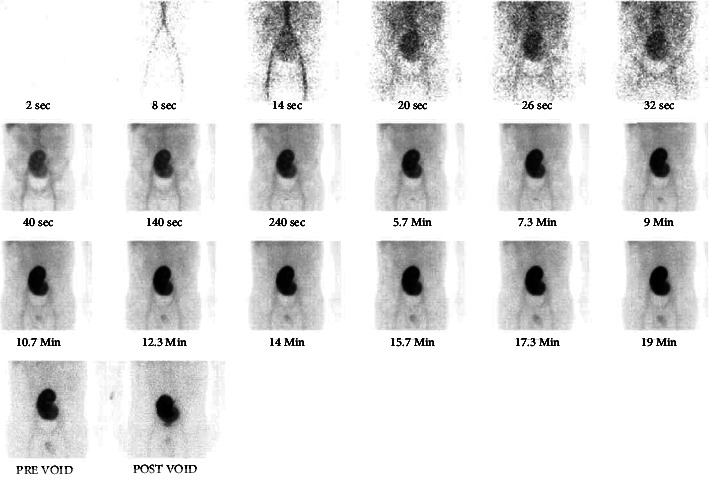
Kidney scintigraphy performed on day 158 post-transplant showing displacement of the transplant kidney to the midline, still with some perfusion present.

He was trialled off dialysis and achieved a baseline creatinine of 250 *μ*mol/L. Pancreatic allograft function remained stable throughout this time. Mobility of the kidney allograft was suspected to be the underlying cause and a semiurgent nephropexy was planned. However, one day prior to his planned surgery, he represented on day 158 post-transplant, again with AKI (creatinine 532 *μ*mol/L from 249 *μ*mol/L two days prior). A third nuclear scan was performed, this time demonstrating reduced perfusion of the kidney allograft with displacement from the left iliac fossa to the central abdomen (Figure 4).

He received a single session of haemodialysis before undergoing urgent surgical correction with nephropexy. This involved surgical fixation of the kidney allograft by creating an extraperitoneal pocket to place the kidney behind the retroperitoneum. A biopsy showed acute tubular necrosis with no evidence of rejection.

The patient has since maintained reasonable allograft function with a baseline creatinine 170 *μ*mol/L (eGFR of 45 mL/min). His pancreatic function remains stable, with no insulin requirement and normal lipase levels. An ultrasound of the pancreatic allograft at 19 months post-transplant revealed that the pseudoaneurysm was not larger at 6.5 × 5.9 mm in size; the smaller pseudoaneurysm could not be visualised. A CT with contrast at 25 months post-transplant demonstrated a pancreatic transplant of normal appearance. Regular imaging surveillance of the pseudoaneurysms will continue at 6–12 monthly intervals, or earlier in the event of symptoms.

## 3. Discussion

This is the first case report to describe two rare complications of SPKT — kidney allograft torsion and pseudoaneurysms of the *Y*-allograft — occurring in one patient.

Kidney torsion is an uncommon cause of allograft failure and requires a high degree of diagnostic suspicion due to its nonspecific symptomatology and variable timing of presentation. At the time of writing, there were26 published cases of kidney transplant torsion, 18 of which involved SPKT with intraperitoneal placement of the kidney. These are summarised in Table 1.

Among the SPKT recipients, for whom the kidney allograft is typically placed intraperitoneally, the timing of the occurrence of kidney allograft torsion was highly variable, ranging from the early postoperative period to ten years post-transplant [7, 11]. Interestingly, of the 8 kidney transplants performed without pancreatic transplantation, 3 of these allografts were placed extraperitoneally and experienced torsion in the early postoperative period [14–16]. Some clinicians have proposed that intraperitoneal placement increases the risk of mobility with subsequent torsion and recommend that prophylactic nephropexy should be performed for patients who require an intraperitoneal kidney transplant [12, 21].

Kidney allograft torsion results in a high rate of allograft loss, with only 58% of allografts salvaged (15 of 26) [7, 8, 11–21]. Successful management depends on rapid diagnosis and surgical correction to limit ischaemic time and, as in our case, can result in a favourable allograft outcome.

Pseudoaneurysm development is a rare complication of SPKT and can present as a medical emergency. Patients may present with rupture leading to significant haemodynamic instability necessitating urgent intervention [22–24]. Asymptomatic presentations such as in our patient are infrequently described [25–28]. Proposed aetiologies for pseudoaneurysm development include infection, vascular damage during transplantation, pancreatitis, and as a complication of biopsy [28–31]. Pseudoaneurysms developing in the setting of previous failed pancreas allografts have also been described [31]. The aetiology of our patient's pseudoaneurysms remains uncertain. We hypothesise that they may have developed as a consequence of vascular injury given the need for a second laparotomy to establish haemostasis.

The current literature guiding management of this uncommon complication comprises case reports and small case series. Most centres reporting their outcomes of pseudoaneurysms describe management via surgical intervention and, in more recent times, an endovascular approach [26, 31–33]. Of the three case reports describing patients with asymptomatic pseudoaneurysms, one patient went on to develop haemorrhage in the setting of anticoagulation and required endovascular intervention, and one underwent surgical exploration due to the large size of the pseudoaneurysm (8 cm) with the final patient declining intervention [26–28]. Our patient, for whom no infective cause for the pseudoaneurysms could be identified, remains asymptomatic following a conservative approach at 40 months post-transplant, and imaging (ultrasound and CT with contrast) indicates that the pseudoaneurysms have remained stable in size, though ongoing surveillance will continue. For our case, conservative management with regular imaging surveillance was felt to be a reasonable option due to the relatively small size of the pseudoaneurysms, lack of clinical symptoms, and absence of an identified aetiology that clearly warranted intervention, such as infection.

In conclusion, kidney allograft torsion, though rare, is an important differential in SPKT recipients with AKI. Swift diagnosis increases the likelihood of allograft salvage, as operative correction is essential. Some transplant specialists recommend prevention via nephropexy [12]. Our case is notable in that there were two significant kidney torsion events both requiring haemodialysis. The first episode resolved spontaneously, and the second required surgical correction.

The occurrence of pseudoaneurysms following SPKT is also unusual, and its management in the literature is variable. Our case is different in a number of aspects, but particularly in our decision to opt for conservative management in light of his asymptomatic presentation.

## Figures and Tables

**Table 1:** Summary table of published cases of kidney allograft torsion.

Case report	Placement of kidney allograft	Transplant type	Timing of torsion	Outcome
Marvin et al. 1995 [5]	Intraperitoneal	Kidney (intraperitoneal adult kidney donor to paediatric recipient)	6 weeks	Transplant nephrectomy
Roza et al. 1998 [6]	Intraperitoneal	2 SPKT^*§*^	11 and 16 months	Transplant nephrectomy
Wong-You-Cheong et al. 1998 [7]	Intraperitoneal	3 SPKT + 2 dual (en bloc) kidney transplants (paediatric donors)	Ranging from 6 to 180 days	2 SPKT + 1 dual kidney transplant had nephropexy; 2 of the 3 were retransplanted The remaining SPKT + dual kidney transplant patients required nephrectomy
West et al. 1998 [8]	Intraperitoneal	4 SPKT	3 weeks, 5 months, 22 months and 2 years	3 allografts salvaged and nephropexy performed, 1 underwent transplant nephrectomy
Badet et al. 2003 [9]	Intraperitoneal	SPKT	7 months	Allograft salvaged and nephropexy performed
Rodrigues et al. 2008 [10]	Intraperitoneal	SPKT	120 days	Transplant nephrectomy
Nangia and Saad 2009 [11]	Intraperitoneal	SPKT	10 years	Allograft salvaged and nephropexy performed
Lucewicz et al. 2011 [12]	Intraperitoneal	Living donor kidney transplant	10 weeks	Transplant nephrectomy
Kaynar et al. 2013 [13]	Intraperitoneal	Living donor kidney transplant + simultaneous bilateral nephrectomies (for polycystic kidney disease)	Two years	Allograft salvaged and nephropexy performed
Ozmen et al. 2013 [14]	Extraperitoneal	Living donor kidney transplant	4 days	Allograft salvaged and nephropexy performed
Winter et al. 2013 [15]	Extraperitoneal	Deceased donor kidney transplant	4 hours	Allograft salvaged, nephropexy not performed
Sosin et al. 2014 [16]	Extraperitoneal	Deceased donor kidney transplant	Day 0	Allograft salvaged and nephropexy performed
Sofue et al. 2015 [17]	Intraperitoneal	2 SPKT	1 month and 3months	Allografts salvaged and nephropexies performed
Dewan et al. 2016 [18]	Intraperitoneal	SPKT	2 years	Allograft salvaged and nephropexy performed
Serrano et al. 2017 [19]	Intraperitoneal	Simultaneous living donor kidney transplant + deceased donor pancreas transplant	3 years	Allograft salvaged and nephropexy performed
Narasimhan et al. 2017 [20]	Intraperitoneal	SPKT	5 weeks	Allograft salvaged and nephropexy performed
Torabi et al. 2018 [21]	Intraperitoneal	SPKT	7 months	Allograft salvaged and nephropexy performed
Tan et al.	Intraperitoneal	SPKT	3 months	Allograft salvaged and nephropexy performed

^*§*^SPKT, simultaneous pancreas-kidney transplantation.

## Data Availability

All data and research outcomes discussed in this study are cited in the Reference section.

## References

[1] ANZOD (2019). *Monthly report on deceased organ donation in Australia*.

[2] Banga N., Hadjianastassiou V. G., Mamode N. (2012). Outcome of surgical complications following simultaneous pancreas-kidney transplantation. *Nephrology Dialysis Transplantation*.

[3] Esmeijer K., Hoogeveen E. K., van den Boog P. J. M. (2020). Superior long-term survival for simultaneous pancreas-kidney transplantation as renal replacement therapy: 30-yearfollow-up of a nationwide cohort. *Diabetes Care*.

[4] Ojo A. O., Meier-Kriesche H. U., Hanson J. A. (2001). The impact of simultaneous pancreas-kidney transplantation on long-term patient survival. *Transplantation*.

[5] Marvin R. G., Halff G. A., Elshihabi I. (1995). Renal allograft torsion associated with prune-belly syndrome. *Pediatric Nephrology*.

[6] Roza A. M., Johnson C. P., Adams M. (1999). Acute torsion of the reanl transplant after combined kidney-pancreas transplant. *Transplantation*.

[7] Wong-You-Cheong J. J., Grumbach K., Krebs T. L. (1998). Torsion of intraperitoneal renal transplants: imaging appearances. *American Journal of Roentgenology*.

[8] West M. S., Stevens B. R., Metrakos P. (1998). Renal pedicle torsion after simultaneous kidney-pancreas transplantation. *Journal of the American College of Surgeons*.

[9] Badet L., Petruzzo P., Lefrancois N., Colombel M., Fassi-Fehri H., Martin X. (2003). Torsion of the renal pedicle after kidney luxation after simultaneous double kidney and pancreas transplantation. *Progrès en Urologie*.

[10] Rodrigues P., Hering F., Gil A. (2008). A well-documented case of chronic renal failure due to misplacement of the transplanted kidney. *Clinics*.

[11] Nangia S., Saad E. R. (2009). Torsion of renal transplant 10 years after simultaneous kidney-pancreas transplantation: imaging as a diagnostic tool. *Transplantation*.

[12] Lucewicz A., Isaacs A., Allen R. D. M., Lam V. W. T., Angelides S., Pleass H. C. C. (2012). Torsion of intraperitoneal kidney transplant. *ANZ Journal of Surgery*.

[13] Kaynar K., Sonmez B., Kutlu O. (2013). A case of recurrent episodes of acute renal allograft failure caused by renal pedicle tortion. *Renal Failure*.

[14] Ozmen M. M., Bilgic I., Ziraman I., Koc M. (2013). Torsion of extraperitoneally transplanted kidney: an unusual complication. *Exp Clin Transplant*.

[15] Winter T. C., Clarke A. L., Campsen J. (2013). Acute torsion of a retroperitoneal renal transplant mimicking renal vein thrombosis. *Ultrasound Quarterly*.

[16] Sosin M., Lumeh W., Cooper M. (2014). Torsion of the retroperitoneal kidney: uncommon or underreported?. *Case Reports in Transplantation*.

[17] Sofue K., Vikraman D. S., Jaffe T. A., Chaubal G. N., Bashir M. R. (2015). Graft kidney torsion after simultaneous kidney-pancreas transplant: report of 2 cases and literature review. *Journal of Computer Assisted Tomography*.

[18] Dewan R., Dasyam A. K., Tan H., Furlan A. (2016). Renal allograft torsion: US and CT imaging findings of a rare posttransplant complication. *Case Reports in Radiology*.

[19] Serrano O. K., Olowofela A. S., Kandaswamy R., Riad S. (2017). Long-term graft survival after kidney allograft torsion: rapid diagnosis and surgical management key to reversibility of injury. *Transplantation Proceedings*.

[20] Narasimhan E., Kennedy A., Campsen J. (2017). Transplant with a twist: a pitfall in sonographic diagnosis of renal transplant torsion. *Journal of Clinical Ultrasound*.

[21] Torabi J., Rocca J. P., Choinski K., Lorenzen K. A., Ajaimy M., Graham J. A. (2018). Renal allograft torsion following simultaneous pancreas kidney transplant should Be suspected with sustained kidney injury with normal pancreas function. *Progress in Transplantation*.

[22] Valle R. D., Capocasale E., Mazzoni M. P. (2005). Embolization of a ruptured pseudoaneurysm with massive hemorrhage following pancreas transplantation: a case report. *Transplantation Proceedings*.

[23] Yiannoullou P., van Dellen D., Khambalia H. (2014). Successful management of a ruptured mycotic pseudoaneurysm following pancreas transplantation using bovine pericardial patch: a case report. *Transplantation Proceedings*.

[24] Akhtar M. Z., Jones A., Sideso E., Sinha S., Vaidya A., Darby C. (2011). Management of a ruptured mycotic pseudo-aneurysm following pancreas-kidney transplantation. *Annals of Transplantation*.

[25] Jaffers G. J., Bohannon W. T., Buckley C. (2011). Endovascular repair of a pancreatic allograft mycotic aneurysm: two-yearfollow-up. *Journal of Endovascular Therapy*.

[26] Lubezky N., Goykhman Y., Nakache R. (2013). Early and late presentations of graft arterial pseudoaneurysm following pancreatic transplantation. *World Journal of Surgery*.

[27] Huurman V. A. L., Lardenoye J. H. P. (2019). Pancreas graft salvage after successful endovascular treatment of Y graft pseudoaneurysm. *Journal of Surgical Case Reports*.

[28] Verni M. P., Leone J. P., DeRoover A. (2001). Pseudoaneurysm of the Y-graft/iliac artery anastomosis following pancreas transplantation: a case report and review of the literature. *Clinical Transplantation*.

[29] Fujita S., Fujikawa T., Mekeel K. L. (2006). Successful endovascular treatment of a leaking pseudoaneurysm without graft loss after simultaneous pancreas and kidney transplantation. *Transplantation*.

[30] Green B. T., Tuttle-Newhall J., Suhocki P., Smith S. R., ’Barry O’Connor J. (2004). Massive gastrointestinal hemorrhage due to rupture of a donor pancreatic artery pseudoaneurysm in a pancreas transplant patient. *Clinical Transplantation*.

[31] Yadav K., Young S., Finger E. B. (2017). Significant arterial complications after pancreas transplantation-Asingle-center experience and review of literature. *Clinical Transplantation*.

[32] Mafeld S., Logue J. A., Masson S. (2019). Treatment of visceral transplant pseudoaneurysms using physician-modified fenestrated stent grafts: initial experience. *CardioVascular and Interventional Radiology*.

[33] Fridell J. A., Johnson M. S., Goggins W. C. (2012). Vascular catastrophes following pancreas transplantation: an evolution in strategy at a single center. *Clinical Transplantation*.

